# Research and Development of* Atractylodes lancea* (Thunb) DC. as a Promising Candidate for Cholangiocarcinoma Chemotherapeutics

**DOI:** 10.1155/2017/5929234

**Published:** 2017-11-14

**Authors:** Kesara Na-Bangchang, Tullayakorn Plengsuriyakarn, Juntra Karbwang

**Affiliations:** ^1^Chulabhorn International College of Medicine, Thammasat University, Phaholyothin Rd, Khlong Luang, Pathum Thani 12120, Thailand; ^2^Clinical Product Development, Nagasaki Institute of Tropical Medicine, Nagasaki University, Nagasaki, Japan

## Abstract

Treatment and control of cholangiocarcinoma (CCA): the bile duct cancer is limited by the lack of effective chemotherapeutic drugs and alternative drugs are needed, particularly those from natural sources. This article reviews steps of research and development of* Atractylodes lancea *(Thunb) DC. (AL) as potential candidate for CCA chemotherapy, with adoption of the reverse pharmacology approach. Major steps include (1) reviewing of existing information on its phytochemistry and pharmacological properties, (2) screening of its activities against CCA, (3) standardization of AL, (4) nonclinical studies to evaluate anti-CCA activities, (5) phytochemistry and standardization of AL extract, (6) development of oral pharmaceutical formulation of standardized AL extract, and (7) toxicity testing of oral pharmaceutical formulation of standardized AL extract. Results from a series of our study confirm anti-CCA potential and safety profiles of both the crude extract and the finished product (oral pharmaceutical formulation of the standardized AL extract). Phases I and II clinical trials of the product to confirm tolerability and efficacy in healthy subjects and patients with advanced stage CCA will be carried out soon.

## 1. Introduction

Cholangiocarcinoma (CCA) is an extremely aggressive cancer with increasing worldwide incidence and mortality rate particularly in Northeastern Thailand [1]. It is a slow-growing ductal adenocarcinoma of biliary epithelial cells lining intrahepatic or extrahepatic bile ducts. The age-standardized incidence rate (ASRs) of CCA in Thailand is 33.4 per 100,000 in males and 12.3 per 100,000 in females. In the UK and USA annual incidence rates are approximately 1-2 cases per 100,000. In Japan, the incidence rate is about 5.5 cases per 100,000 [2]. The major risk of CCA in most countries is the primary sclerosing cholangitis, but in Asian countries the major risk factor is liver fluke infection (*Opisthorchis viverrini* and* Clonorchis sinensis*).

With early detection, surgical resection is the treatment of choice but there is a lack of effective early diagnostic tools [3]. Most patients with CCA are diagnosed at late stage and surgical resection is no longer possible [4]. The efficacy of existing chemotherapy and radiotherapy are also limited to patients with early stage of the disease. Furthermore, the cancer cells are developing resistance to both interventions [5, 6]. Treatment efficacy of the standard treatment with 5-fluorouracil (5-FU), gemcitabine, or their combinations with cisplatin is unsatisfactory with low clinical response rate [7]. Less than 5% of the advanced stage patients survive for up to five years [8]. The primary reason for disease poor prognosis is metastasis, which precludes curative surgical resection. Advanced surgical techniques in conjunction with alternative chemotherapeutic option with promising activity are required to improve the survival of patients [9]. There is a need to search for the treatment that is active against both the late stage of the CCA and metastasis.

**Figure 1:**
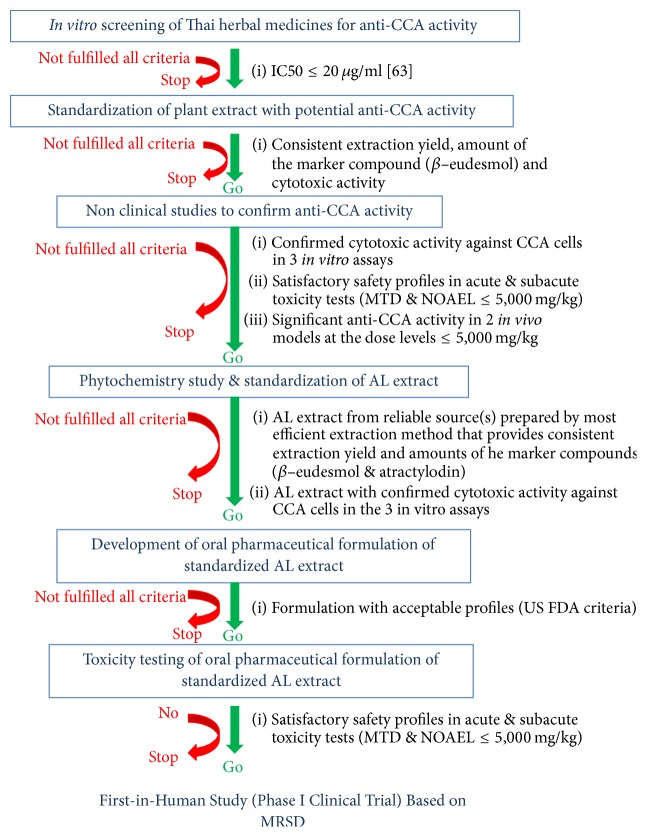
A diagram summarizing criteria for decision making of go or no-go for each step at discovery and preclinical phases of AL development. The right column is set of criteria during each step (IC_50_ is concentration that inhibits cell growth by 50%; MTD is maximum tolerated dose; NOAEL is no observed adverse effect level; MRSD is maximum recommended starting dose).

The use of natural products as complementary and alternative medicine has been increased dramatically in the last decades. Natural products, in particular herbs, constitute a promising approach for the discovery and development of new drug candidates including for cancer chemotherapy. This is due to the diverse chemical structures and bioactivities of herbs against several health disorders. The previously reported studies of various potential plants for CCA have been focused solely on antiproliferative activities of plant extracts or isolated compounds on CCA cell lines or animal models, or on their underlying mechanisms of action on cell apoptosis and cell cycle arrest [10–23]. However, none of these studies has undergone full processes of development to obtain final standardized products for clinical uses. In the past few years, we have performed series of studies focused on the research that support the development of* Atractylodes lancea* (Thunb) DC. (AL) as a potential chemotherapeutic for CCA. The aim of this article is to describe the results of a series of research of natural product that are required to support further development of AL as treatment of CCA. We have adopted the reverse pharmacology approach, starting from (1) reviewing of existing information on its phytochemistry, ethnopharmacology, and pharmacological properties, (2) screening of its activities against CCA, (3) standardization of AL extract, (4) nonclinical studies to evaluate anti-CCA activities, (5) phytochemistry and standardization of AL extract, (6) development of oral pharmaceutical formulation of standardized AL extract, and (7) toxicity testing of oral pharmaceutical formulation of standardized AL extract. A diagram summarizing criteria for decision making of go or no-go for each step is shown in Figure 1.

Despite the complex nature of the herbal product, rigorous methodologies of scientific and clinical standards were applied to fulfill the requirements of regulatory agencies (FDA) throughout all steps of development to prove the safety and efficacy of the product. To optimize the reproducibility of all studies, the plant extract was sufficiently characterized and standardized using key constituents as biomarkers for quality control of the finished product. Large scale pharmaceutical formulation of standardized AL extract for oral use is being prepared for phase I and phase II clinical trials.

## 2. Review of the Literatures on* Atractylodes lancea *(Thunb) DC. 

### 2.1. Phytochemistry

**Figure 2:**
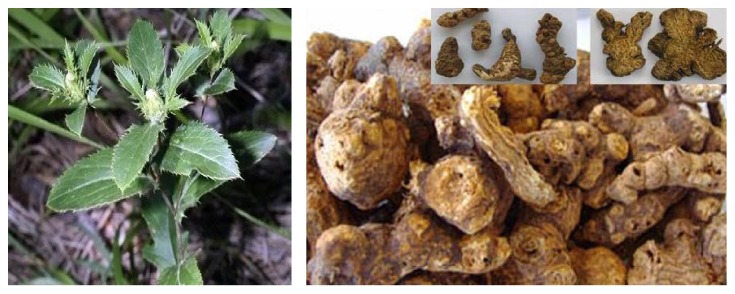
*Atractylodes lancea* (Thunb) DC. (leaves and rhizomes).

**Figure 3:**
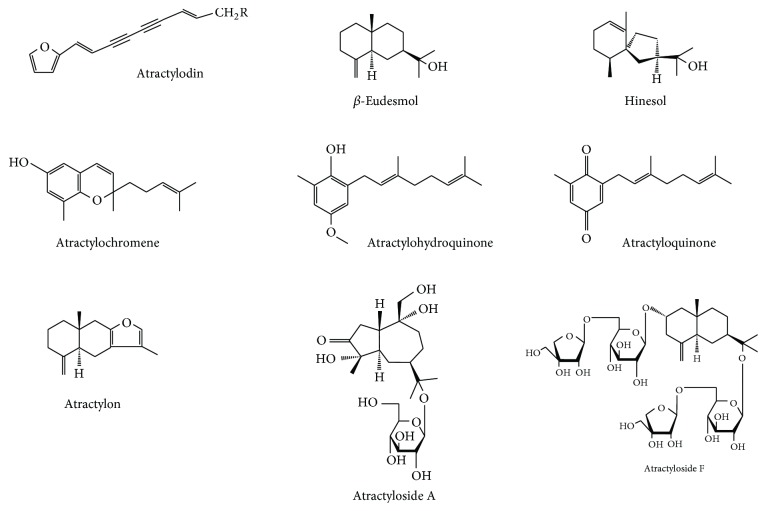
Chemical structures of isolated compounds from* Atractylodes lancea *(Thunb) DC. (modified from Ouyang et al., 2007 [24]).

*Atractylodes lancea* (Thunb) DC. is widely distributed in East Asia, particularly in China (e.g., Anhui, Hubei, and Jiangsu). It is a member of plant in the family Asteraceae (Compositae). Its rhizome (3–10 cm) appears as yellowish-brown color (Figure 2). AL rhizome is rich in volatile oil, making up of approximately 3.5–7% of the dried rhizomes. Previous phytochemical investigations revealed a series of sesquiterpenoids, monoterpenes, polyacetylenes, phenolic acids, and steroids from AL rhizomes [28–35]. The major constituents include atractylodin (14%), *β*-eudesmol (6%), atractylon (2%), and hinesol (1%) (Figure 3). Other minor constituents include atractyloside A, atractyloside F, atractyloquinone, and atractylochromene (Figure 3) [24, 36].

### 2.2. Ethnopharmacology and Pharmacological Properties

The dried rhizome of* A. lancea *(AL) is a common medicinal plant used in traditional Chinese medicine (“Cang Zhu”), and Japanese campo (“So-jutsu”), as well as in Thai traditional medicine (“Khod-Kha-Mao”). In Chinese traditional medicine, AL has been used as an important crude drug for treatment of rheumatic diseases, digestive disorders, night blindness, and influenza [37, 38] and was listed in “Shen-nong-ben-cao-jing,” the first Chinese pharmacopoeia [39]. In the Korean and Japanese pharmacopoeias, the rhizome of AL has been prescribed in traditional medicine as diuretic and stomachic drugs [35]. In Thai traditional medicine, the main use of AL is for treatment of fever and cold [40]. These ethnopharmacological uses of AL either as single extract or as a composition in herbal medicine formula are explained by its properties in eliminating dampness, vigorating spleen, and expelling wind according to the theory of Chinese traditional medicine.

Modern pharmacological studies support broad pharmacological effects of AL on various diseases. The pharmacological activities of AL rhizomes have previously been reviewed [41].* In vitro* and* in vivo* studies in animal models demonstrate promising activities of the crude extracts (ethanolic, water, benzene, and methanol) of AL rhizomes as well as its major constituents (*β*-eudesmol, atractylodin, hinesol, atractylon, atractykochromene, and quinone) on cancers, inflammation, fever and pain, gastrointestinal system, nervous system, cardiovascular system, and various microorganisms (*Staphylococcus aureus, Escherichia coli, Saccharomyces cerevisiae, Candida albicans, Rhodotorula glutinis*, and* Saprolegnia*). In addition, hepatoprotective and immunostimulating activity of AL have also been demonstrated [42–44]. The pharmacological activities of AL and its constituents on gastrointestinal system (delay of gastric emptying, stimulation of intestinal motility, inhibition of gastric secretion, and antiulcer property) strongly support their clinical use for alleviation of digestive symptoms in traditional medicine [25, 45–47]. AL was also shown to increase the levels of gastric hormone motilin and gastrin, while decreasing the levels of somatostatin and corticotrophin-releasing factor, which results in improving the gastric emptying condition [48, 49]. The traditional use of AL for anti-inflammatory effect is demonstrated to be due mainly to interference with the enzymes in the inflammatory pathway, that is, 5- lipoxygenase (5-LOX) and cyclooxygenase-1 (COX-1) [33]. It is important however for the knowledge of potential deleterious effects of AL on cardiovascular system. The antiplatelet aggregation of AL is possibly mediated through suppression of collagen-induced signaling pathway which is the upstream of the release of thromboxane A2 from platelets [50]. Care should be taken when using AL extract or its active constituents in patients with platelet disorders or coagulopathy. Although neither serious adverse effect on central nervous system nor any morbidity has been reported in human so far, the clinical use of AL should also be with caution in patients with nervous problems due to its effects on various organ systems (general behavior and spontaneous movement, potentiation of hypnotic action of hexobarbital, and interference with muscle relaxation) [25, 51–56]. The neurological activity of *β*-eudesmol, including its antagonistic effect on neostigmine-induced neuromuscular failure, was demonstrated in previous studies on the accelerating effect of desensitization of the nicotinic acetylcholine receptor and on blockage action during the closed state of the receptor channel or nerve-evoked contraction at the neuromuscular junction [55].

The potential anticancer and antiangiogenesis properties of AL extract and its major constituents have been demonstrated in various cancer, for examples, murine blastoma cells Hela (human cervical cells), SGC-7901 (human gastric cancer cells), BEL-7402 (human liver cancer cells), H33, S180, HL-60 leukemic cells, and gastric cancer [57–61].

## 3. Screening of Plants Used in Thai Traditional Medicine as Potential Sources of Drugs to Control Cholangiocarcinoma

In an effort to identify Thai medicinal plants with potential anti-CCA activities, the ethanolic extracts of a total of 28 plants and 5 recipes used in Thai folklore medicine were initially screened for their cytotoxic activities against the human CCA cell line CL-6 [62]. In addition, their comparative activities against Hep-2 (laryngeal carcinoma), HepG2 (hepatocarcinoma), and HRE (human normal renal epithelial cell) cell lines were also examined. The extracts from the five plants, that is,* Atractylodes lancea* (rhizome),* Kaempferia galanga *(leaf),* Zingiber officinal* (rhizome),* Piper chaba* (fruit),* Mesua ferrea* (flower), and Pra-Sa-Prao-Yhai recipe (a mixture of 18 plants including* A. lancea*) showed potent cytotoxic activity against CL-6 cell line with mean IC_50_ (concentration that inhibits cell growth by 50%) values of 24.09, 37.36, 34.26, 40.74, 48.23, and 44.12 *μ*g/ml, respectively. Based on the criteria of the US National Cancer Institute Plant Screening Program, a crude extract is generally considered to have* in vitro* cytotoxic activity if the IC_50_ value in carcinoma cells is less than or at least equal 20 *μ*g/ml [63]; only the extract from AL was shown to be the most potent and selective against CCA (selectivity index, SI: the ratio of IC_50_ of the AL extract in CL-6 cell and that in HRE cell = 8.6). It is considered more potent and selective to CCA cell than the reference drug 5-FU (5-fluorouracil: IC_50_ = 98.41 *μ*g/ml, SI = 2.0). In addition, AL also exhibited potent and selective activity against Hep-2 (mean IC_50_ = 29.35 *μ*g/ml, SI = 7.1) and moderate activity against HepG2 (mean IC_50_ 76.68 *μ*g/ml, SI = 2.7) cells.

## 4. Standardization of* Atractylodes lancea *(Thunb) DC. Extract

### 4.1. Plant Source and Preparation

Preparation of the rhizome extract of AL was standardized for further detailed investigations to confirm potential anti-CCA activity and safety profile both* in vitro* and* in vivo*. The rhizomes were supplied by a local traditional pharmacy “Charoensuk Osod” in Nakhon Pathom province, Thailand, of which the original source was from Heilongjiang, China. Authentication of the plant materials (voucher number SKP 051011201) was performed (macroscopic and microscopic characterization) at the herbarium of the Department of Forestry, Bangkok, Thailand, where the herbarium voucher was archived. The AL rhizomes were rinsed thoroughly with tap water and cut into small pieces, oven dried at 50°C, and ground into powder. The samples were pulverized, kept in sealed plastic bags, and stored at room temperature (25°C).

### 4.2. Preparation of Crude Extract

**Figure 4:**
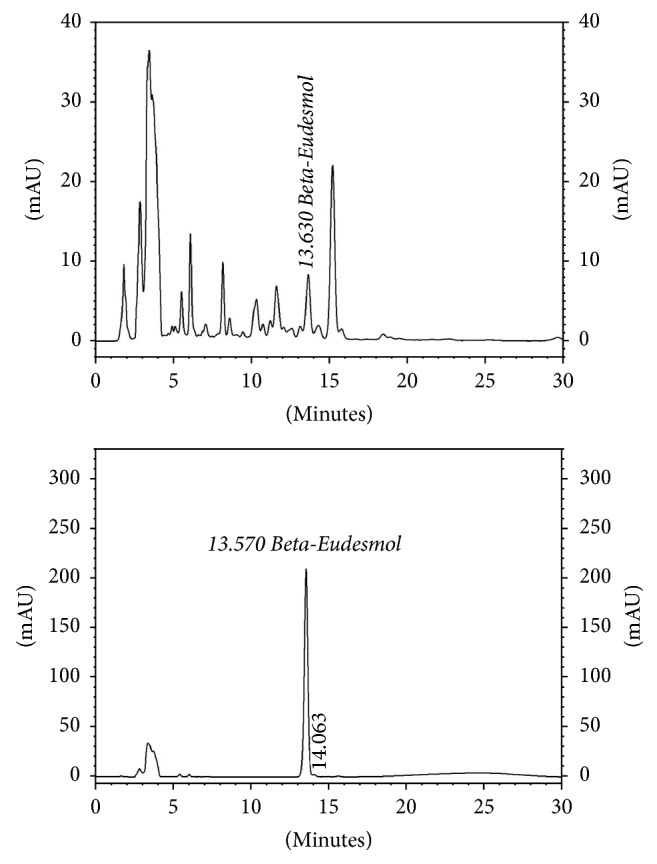
Standardization of the ethanolic extract of* Atractylodes lancea* (Thunb) DC. (rhizomes) by high performance liquid chromatography using *β*-eudesmol as a marker for quality control.

The AL extract was prepared by macerating the powdered AL rhizome (100 g) in a stopper flask containing 500 ml of 95% ethanol at room temperature (25–30°C) for 7 days [62]. The extracted solvent was separated and filtered through Whatman number 1 filter paper (GE Healthcare, Maidstone, UK). After filtration, the extract was evaporated under reduced pressure by rotary evaporation. The dried powder was weighed and stored at −20°C until use. Quality control of the extract was performed using high performance liquid chromatography (HPLC) to determine relative amount of *β*-eudesmol (marker compound) [27]. Chromatographic separation condition used was as follows: Thermo Hypersil Gold C18 column (Thermo Scientific, Rockford, IL, USA) and mobile phase consisting of a mixture of water and acetonitrile with isocratic elution of 40 : 60 (v : v) at the flow rate of 1 ml/min and run time of 30 min. The wavelength was set at 203 nm. The extract yield was 16.89% with relative amount of *β*-eudesmol of 6.73 (Figure 4).

## 5. Nonclinical Studies to Investigate Anticholangiocarcinoma Potential and Safety of* Atractylodes lancea* (Thunb) DC. 

### 5.1. Cytotoxicity, Antioxidant, and Clonogenic Survival Activities and Inhibitory Activities on Angiogenesis and Cell Invasion

A series of* in vitro* experiments was conducted to confirm the potential anti-CCA activity of AL [64]. These included cytotoxic, antioxidant, and inhibitory activities on angiogenesis and cell invasion. AL was shown to produce significantly greater potency of cytotoxic, anticlonogenic, and inhibitory activities on cell invasion compared with the reference drug 5-FU. The antiangiogenic activity was comparable to 5-FU.

Cytotoxic effect of AL extract on human CCA CL-6 cells was confirmed in two additional assays beside the MTT assay (measures the effects of test materials on mitochondria), that is, Calcein-AM release assay (indirectly measures cell esterase activity) and Hoechst 33342 assay (measures cell DNA binding). In both assays, AL was found to inhibit CL-6 cell viability in a concentration-dependent manner following 48-hour exposure. The mean IC_50_ of AL including their SI values in both assays were 23.24 *μ*g/ml versus 10.98 and 27.16 *μ*g/ml versus 10.16, respectively. The cytotoxic activity of AL against CL-6 cells including its selectivity based on both assays were about 4 and 3 fold of 5-FU, respectively. The cytotoxic effect of AL was more specific to CL-6 cells compared with HepG2 (SI = 5.28 and 6.36 for calcein-AM and Hoechest 33342 assay, respectively). Reproductive toxicity of AL was also well demonstrated in the clonogenic survival assay with potent activity compared with 5-FU (98% and 52% at 100 *μ*g/ml, respectively). AL produced weak antioxidant activity with potency of about 9 times of ascorbic acid (reference compound). The IC_50_ (mean ± SEM) values of the antioxidant activities of AL and ascorbic acid were 16.95 ± 0.35 and 154.78 ± 0.84 *μ*g/ml, respectively. This activity may serve as one of the underlying mechanisms of anti-CCA action of AL on interfering with free radical mediated oxidative stress in CCA tumor genesis [65].

Metastasis (spread of tumor cells to other tissues through cell invasion) and angiogenesis (a process of new blood vessel development) are the two important characteristic features of malignant tumors including CCA. The incidence of lymph node and remote organ metastasis were reported to be 75% and 71%, respectively [66]. CL-6 cell invasion was significantly reduced following exposure to AL and 5-FU in a concentration-dependent manner. The potency of activity was about 1.5 fold of 5-FU. The effect of AL on angiogenesis was evaluated by examining its inhibitory effect on the development of tube formation of endothelial cells using Angiogenesis Assay kit. AL at 25, 50, and 100 *μ*g/ml significantly inhibited tube formation of endothelial cells by 56%, 84%, and 100% of control, respectively. The potency of activity at all concentrations was about 1.2 fold of 5-FU.

### 5.2. Toxicity Testing (Acute and Subacute Toxicity)

Acute (single oral dose) and subacute (daily oral doses for 28 days) toxicity testing of the crude ethanolic extract of AL were carried out in mice, rats, and hamsters to find maximum tolerated dose (MTD) and no observed adverse effect (NOAEL) levels of AL to be used for anti-CCA evaluation [25, 26]. The experiments were performed in accordance with the OECD guideline for testing of the chemicals [67, 68]. In all animal species, AL shows virtually no toxicity up to the highest recommended dose of 5,000 mg/kg body weight. No animal died during the observation period. Only mild signs of acute toxicity occurred in all animals receiving highest dose level of AL (5,000 mg/kg body weight). These included stomach irritation and general CNS depressant signs (reduced alertness and locomotion and diminished response to touch and balance) which occurred within 1 hour after feeding with the extract. Stomach irritation was observed in all animals immediately after feeding. All symptoms subsided within 2 hours of administration. No histopathological abnormality was observed in any vital organ at autopsy. Both MTD and NOAEL dose levels of AL in acute and subacute toxicity testing of AL extract in all animal species were 5,000 mg/kg body weight.

In addition to the crude AL extract, acute and subacute toxicity of *β*-eudesmol was also evaluated in mice in order to find the MTD and MOAEL levels for further investigation of its anti-CCA activity and pharmacokinetics in CCA-xenografted nude mouse model [27]. The MTD and NOAEL dose levels of *β*-eudesmol were 100 mg/kg body weight. The lethal dose (LD) was 200 mg/kg body weight.

### 5.3. *In Vivo* Anticholangiocarcinoma Activity

Several animal models were used to investigate the anti-CCA activities of candidate compounds or medicinal plant extracts including xenograft and orthotopic models, carcinogen-induced CCA model, and genetically engineered mouse model [69–71]. The CCA-xenografted mouse model and* Opisthorchis viverrini*/dimethylnitrosamine-induced CCA hamster model were used in our studies to confirm the anti-CCA potential of AL and *β*-eudesmol. The advantage of the first is its simplicity and ease of monitoring of tumor progression. The second model, however, more closely mimics the pathogenicity of human CCA.

#### 5.3.1. CCA-Xenografted Nude Mouse Model

**Figure 5:**
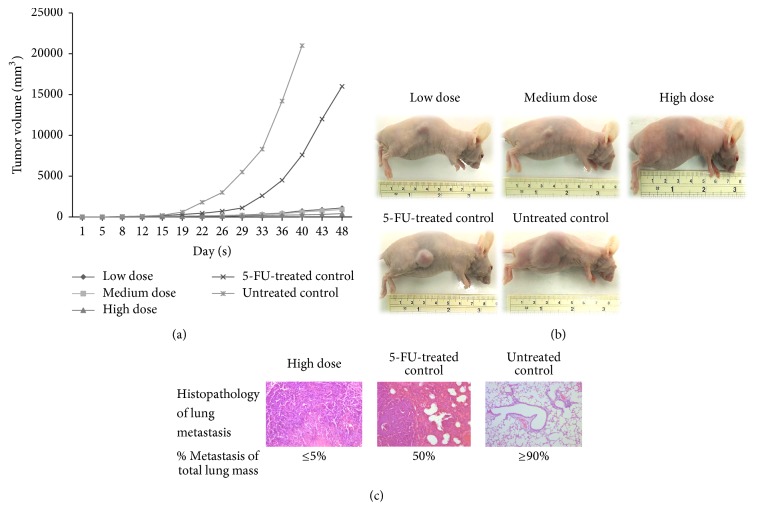
Anti-CCA activity of AL extract [low dose (1,000 mg/kg body weight), medium dose (3,000 mg/kg body weight), and high dose (5,000 mg/kg body weight)], 5-FU and untreated control in CCA-xenografted nude mice; (a) line graphs showing development of tumor volume, (b) tumor mass of mice, and (c) lung metastasis (modified from Plengsuriyakarn et al., 2012 [25]).

Anti-CCA activity of the ethanolic extracts of AL was initially evaluated in CCA-xenograft nude mouse model [25]. The human CCA cell line CL-6 was implanted into this subcutaneous of the nude mice which lack immunity. AL produced significant anti-CCA activity compared with 5-FU and untreated control. AL at all dose levels (oral daily dose for 30 days), that is, low (1,000 mg/kg body weight), medium (3,000 mg/kg body weight), and high (5,000 mg/kg body weight) dose levels, significantly inhibited tumor growth to about 2% of the untreated control, while 5-FU inhibited tumor growth to only 70% of the control (Figures 5(a) and 5(b)).

Metastasis is one of the major problems in the treatment of CCA. In advanced stage, metastatic lung tumors develop by spreading of CCA from the liver origin through the bloodstream or lymphatic system (less than 5% of lung metastasis). Interestingly, significant reduction of lung metastasis was observed in the xenografted mice treated with AL compared with untreated control and 5-FU (Figure 5(c)). Lung metastasis (expressed as mean percentage of total lung mass) following treatment with 5,000, 3,000, and 1,000 mg/kg body weight AL, 5-FU, and untreated control were 5%, 25%, 70%, 50%, and 90%, respectively. Histopathological findings revealed prominent inhibition of lung metastasis by AL at the highest dose of 5,000 mg/kg body weight. This observation corresponds well with the results of its inhibitory activity on cell invasion and angiogenesis assays as described above [64]. The hepatocyte growth factor HGF/Met has been shown in a previous study to play a role in cell invasion by promoting CCA cell invasiveness through dyslocalization of E-cadherin and induction of cell motility by distinct signaling pathways [72]. Moreover, revision-inducing-cysteine-rich protein with Kazal motifs (RECK) has been implicated in the attenuation of CCA tumor metastasis by negatively regulating metalloproteinase levels [73]. Further investigations into mechanisms of inhibitory effect of AL on these molecular targets in CCA metastasis and angiogenesis processes are underway.

#### 5.3.2. *Opisthorchis viverrini*/Dimethylnitrosamine-Induced CCA Hamster Model

**Figure 6:**
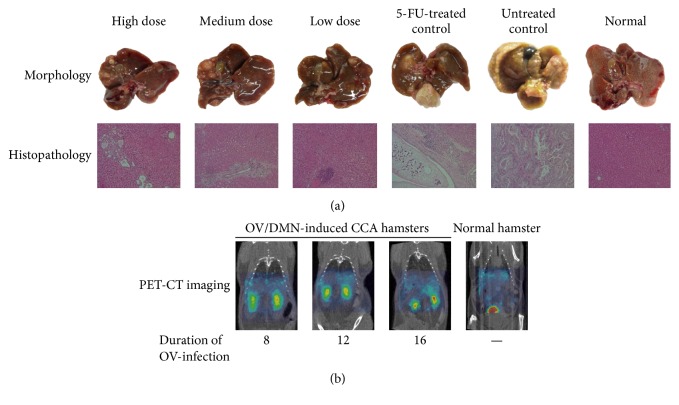
Anti-CCA activity of AL extract [low dose (1,000 mg/kg body weight), medium dose (3,000 mg/kg body weight), and high dose (5,000 mg/kg body weight)], 5-FU and untreated control in* Opisthorchis viverrini*/dimethylnitrosamine-induced CCA hamsters; (a) gross morphology and histopathology of livers and (b) representative PET-CT images of CCA (modified from Plengsuriyakarn et al., 2015 [26]).

The anti-CCA activity of AL was further confirmed in an additional model of* Opisthorchis viverrini* (OV)/dimethylnitrosamine- (DMN-) induced CCA hamster model [26]. Development of CCA was induced by initial feeding of male hamsters (by gastric gavage) with 50 metacercariae of OV, followed four weeks later by drinking water containing 12.5 ppm of DMN for eight weeks. The study was the first study that applied positron emission tomography-computed tomography (PET-CT) imaging with radio tracer ^18^F-FDG as a tool for detection and monitoring the progression of CCA throughout the observation period. Histopathological examination of all OV/DMN-induced CCA hamsters confirmed the occurrence of CCA with proliferation and cystic formation of bile duct and infiltration of inflammatory cells (lymphocytes, plasma cells, and macrophages) around portal areas (Figure 6).

Similarly to that observed with the CCA-xenografted mouse model [25], promising anti-CCA activity of AL was observed at all dose levels, particularly at the highest oral dose level of 5,000 mg/kg body weight for 30 days. The histopathological lesion of the bile ducts was markedly less pronounced in the AL-treated hamsters compared with 5-FU treated and untreated control; only mild diffuse proliferation, cystic formation, and fibrosis of bile ducts were found (Figure 6). The median survival rate and survival time were significantly prolonged (about 2 times) in hamsters treated with the extract at all dose levels compared with 5-FU treated and untreated control groups during the 4–6 months observation period. At week 36, all hamsters except those treated with the highest AL dose died (1 hamster died, 80% survival rate). The untreated control animals started to die as early as 14 weeks.

### 5.4. Other Pharmacological Activities

The pharmacological activities (motor coordination, antihypertension, analgesia, anti-inflammation, antiulcer, and antipyrexia) of the ethanolic extract AL were investigated in various* in vivo* models [25]. The antihypertensive, analgesic, anti-inflammatory, antipyretic, and antiulcer activities and effects on motor coordination were examined using Rota-rod test, CODA tail-cuff system, writhing and hot plate tests, carrageenan-induced paw edema test, brewer's yeast test, and alcohol-induced gastric ulcer test, respectively. No significant effect of AL on motor coordination including antihypertensive and analgesic activities was observed at all dose levels (1,000, 3,000, and 5,000 mg/kg body weight). On the other hand, highest AL dose (5,000 mg/kg body weight) significantly produced anti-inflammatory and antipyretic activities compared with reference drugs (indomethacin and aspirin for anti-inflammatory and antipyretic activity, resp.) and untreated control. The anti-inflammatory activity of high dose AL, indomethacin (10 mg/kg body weight), and untreated control were 33.3%, 40.7%, and 0%, respectively. The antipyretic activity of high dose AL, aspirin (200 mg/kg body weight), and untreated control were 80%, 100%, and 0%, respectively. The compound 2-[(2′E)-3′,7′-dimethyl-2′,6′-octadienyl]-4-methoxy-6-methylphenol isolated from AL rhizome has been shown to potently inhibit 5-LOX and COX-1 [30], the two key enzymes which play a central role in the regulation of different physiological and pathological processes including fever, pain, inflammation, and hypersensitivity.

For antiulcer activity, significant reduction (96.47–98.24%) of ulcer size was observed in rats pretreated with all dose levels of AL compared with the reference drug (20 mg/kg body weight omeprazole) (93.04%) and untreated control (0%). The observed significant antiulcer activity of AL confirms its use to improve stomach damage partly through antiulcer effects [46].

## 6. Phytochemistry and Standardization of* Atractylodes lancea *(Thunb) DC. Extract

Phytochemistry investigation and standardization of AL extract were performed to ensure the identity (variation in sources) and quality of the AL extract to be used in further steps. These steps included nonclinical studies of pharmacological (anti-CCA and pharmacokinetics) and toxicological profiles of AL, large scale production of oral pharmaceutical formulation of standardized AL extract, and clinical trials to evaluate efficacy, tolerability, and pharmacokinetics of AL.

### 6.1. Plant Materials and Selection of* Atractylodes lancea* (Thunb) DC. Supply Source

AL rhizomes used in the study were supplied by the two local traditional pharmacies in Thailand, that is, the traditional herbal pharmacies in Bangkok (Chaokrompoe) and Nakhon Pathom (Charoensuk Osod). The origin of plant source supplied by the first shop was unknown, whereas that supplied by Nakhon Pathom was originally from Heilongjiang, China. Authentication of the plant materials (voucher number SKP 051011201) was performed at the herbarium of the Department of Forestry, Bangkok, Thailand, where the herbarium voucher was archived. Preparation of the plant materials and storage condition has been described in Section 4.1.

The ethanolic extracts of AL obtained from the two sources were compared for their extraction yield and cytotoxic activities against the CCA CL-6 cells using MTT assay. The extract from each source was prepared using maceration method as previously described in Section 4.2. Quality control of the extracts was evaluated based on relative amounts of the marker compound *β*-eudesmol and in addition, atractylodin using HPLC (Figure 6). The chromatographic condition used was as follows: Thermo Hypersil BDS C18 column and mobile phase consisting of a mixture of water and methanol with isocratic elution of 50 : 50 (v : v) running for 112 min at the flow rate 1 ml/min. The wavelength was set at 203 nm. Results showed that the AL extract from Charoensuk Osod herbal pharmacy provided the highest yield (10.74%) and maximum cytotoxic activity (IC_50_ of 33.15 *μ*g/ml) compared with that from Chaokrompoe herbal pharmacy (5.56% yield and IC_50_ 61.25 *μ*g/ml). The selectivity of cytotoxic activity toward CCA (compared with human embryonic fibroblast cell OUMS-36T) of the extract obtained from both sources was comparable (SI of 3.28 and 1.86, resp.). Based on the US National Cancer Institute criteria [74] and consideration of the known original source of AL, Charoensuk Osod herbal pharmacy was selected as a reliable supply source of AL rhizomes for further studies and large scale production of the oral pharmaceutical formulation of standardized AL extract.

### 6.2. Selection and Optimization of Extraction Method

**Figure 7:**
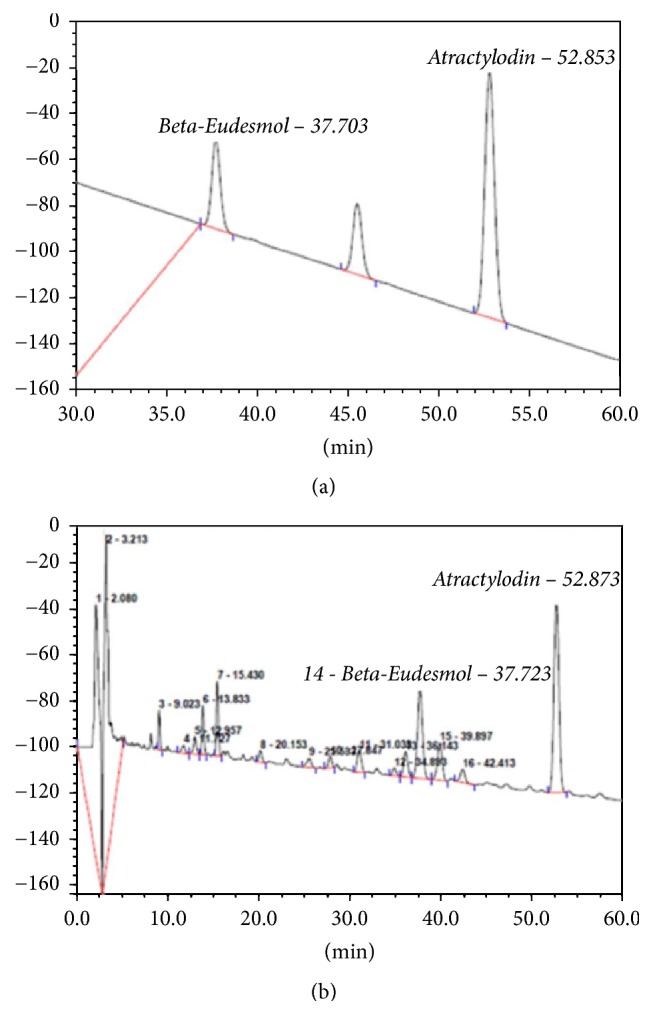
Standardization of the ethanolic extract of* Atractylodes lancea* (Thunb) DC. (rhizomes) by high performance liquid chromatography using *β*-eudesmol and atractylodin as markers for quality control.

Three different extraction methods, that is, maceration (95% ethanol, 25–30°C, 7 days), sonication (95% ethanol, 25–30°C, 30 minutes), and heat reflux (95% ethanol, 95°C, 50 minutes), were initially used to prepare AL ethanolic extract with maximum extraction efficiency (% yield) and cytotoxic activity. Quality control of the extracts was evaluated based on relative amounts of the marker compound *β*-eudesmol and atractylodin using the previously described HPLC condition (Figure 7). The extraction efficiencies (% yields) of different methods in descending order were heat reflux (12.10%), followed by sonication (11.90%), and maceration (11.75%).

The cytotoxic activities of the AL extracts prepared by the three methods and 5-FU against CL-6 were evaluated using MTT assay. All methods provided the extracts with comparable cytotoxic activities against CL-6 (mean ± SD IC_50_: 33.02 ± 0.18, 26.15 ± 0.47, 33.614.70 *μ*g/ml, and 107.72 ± 1.69 *μ*g/ml, resp.). The potency of activities of the AL extracts was about 3.3–4.2 fold of 5-FU.

The maceration method, although not the most efficient method, was selected for further optimization due to its simplicity, cost-effectiveness, and applicability to large scale production. Four maceration conditions were investigated, that is, single maceration for 24 hours, single maceration for 72 hours, double maceration (24 hours each), and triple maceration (24 hours each). Cytotoxic activity against CL-6 of the extract from each maceration condition was evaluated using MTT assay. The most efficient maceration condition which provided the highest extraction efficiency (10.74% yield) was triple maceration for the duration of 24 hours. The maceration condition which provided the extract with most potent cytotoxic activity (IC_50_  27.49 ± 0.98 *μ*g/ml) was single maceration for the duration of 24 hours. Considering the compromised performance of the extraction efficiency and cytotoxic activity, double maceration for 24 hours was selected as the most optimal condition for preparation of the AL extract for nonclinical and clinical studies and large scale production of the pharmaceutical formulation.

## 7. Nonclinical Studies to Evaluate Anticholangiocarcinoma Activities of the Bioactive Compounds from* Atractylodes lancea* (Thunb) DC. 

### 7.1. Cytotoxic Activities of the Bioactive Compounds and Their Interactions When Used in Combination

The cytotoxic activities of the four main bioactive compounds from* Atractylodes lancea*, that is, atractylodin (14%), *β*-eudesmol (6%), hinesol (1%), and atractyloside III (less than 1%) were investigated in CL-6 cells using MTT assay (manuscript in preparation). In addition, their interactions on cytotoxic activities against CL-6 cells when used in combination were also examined by isobologram/polygonogram analysis. *β*-Eudesmol and atractylodin potently inhibited CL-6 cell growth with mean (±SD) IC_50_ of 21.5 ± 2.12 and 24.0 ± 1.98 *μ*M, respectively. The cytotoxic activities of atractyloside III and hinesol were relatively weak [with mean (±SD) IC_50_ of 145.9 ± 14.4 and 91.0 ± 8.0 *μ*g/ml, resp.]. The interaction between *β*-eudesmol and atractylodin was shown to be additive (mean sum fractional inhibitory concentration: FIC = 0.998–1.21). On the other hand, the interactions between *β*-eudesmol and hinesol, atractylodin and hinesol, and *β*-eudesmol and atractylodin and hinesol were synergistic (mean sum FIC of 0.35–0.84, 0.411–0.58 and combination index (CI) of 0.519–0.65, resp.). Results of this experiment support the previously described results on the cytotoxic activity of each compound against CL-6 cells and, most importantly, the decision to use crude AL extract for evaluating potential anti-CCA activity both in animals and humans.

### 7.2. Anti-CCA Activity of *β*-Eudesmol in CCA-Xenografted Nude Mouse Model

**Figure 8:**
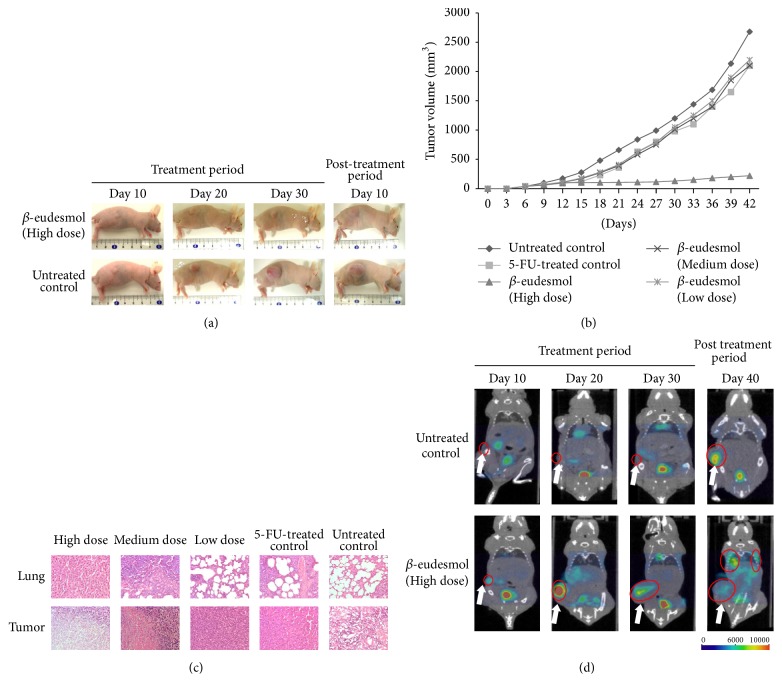
Anti-CCA activity of *β*-eudesmol and 5-FU in comparison with untreated control in CCA-xenografted nude mice; (a) development of tumor mass tumor, (b) tumor growth curves, (c) histopathology of lung metastasis, and (d) representative PET-CT images of CCA (modified from Plengsuriyakarn et al., 2015 [27]).

Based on results of the cytotoxic activity of *β*-eudesmol described above, together with its evidence on antiangiogenesis and antimetastasis activities [27, 58], *β*-eudesmol was selected as a promising compound for further evaluation of the pharmacokinetics of AL. The anti-CCA activity of the bioactive compound *β*-eudesmol was investigated in a CCA-xenografted nude mouse model at three dose levels, that is, high (100 mg/kg body weight), medium (50 mg/kg body weight), and low (10 mg/kg body weight) given by daily oral dose for 30 days [27]. PET-CT with ^18^F-fluorodeoxyglucose (^18^F-FDG) was used for detecting and monitoring tumor development in healthy and CCA-xenografted nude mice. High dose *β*-eudesmol potently inhibited tumor growth and prolonged survival time of mice compared with 5-FU and untreated control (Figures 8(a) and 8(b)). Median (95% CI) tumor volumes on day 42 of mice treated with *β*-eudesmol, 5-FU, and untreated control were 212 (195–235), 2,109 (2,100–2,117), and 2,556 (2,465–2,646) mm^3^, respectively. The corresponding values for survival time were 71.5 (70.3–72.2), 54.5 (52.6–56.4), and 43.5 (42.3–44.7) days, respectively. The survival time of mice was prolonged by 64.4% compared with the untreated control. Tumor size and lung metastases were significantly inhibited by 91.6% (of baseline) and 95% (of total lung mass), respectively. The inhibitory activity of *β*-eudesmol on lung metastases was evidently demonstrated both morphologically and histopathologically (Figure 8(a)), with confirmation by results of PET-CT imaging in CCA-xenografted mice (Figure 7(b)). Results of potent anti-CCA activity of AL together with its antimetastasis and antiangiogenic properties supported the findings in OV/DMN-induced CCA hamster model and CCA-xenografted nude mouse model as described in Sections 5.3.1 and 5.3.2 [25, 27].

### 7.3. Mutagenic Activities of Ethanolic Extract of* Atractylodes lancea* (Thunb) DC., *β*-Eudesmol, and Atractylodin in Cholangiocarcinoma Cell Lines

The effects of AL ethanolic extract, *β*-eudesmol, and atractylodin on mutagenesis were investigated in the CCA cells CL-6 and HuCCT-1 as well as normal cell line OUMS-36T-1F using micronucleus assay (manuscript in preparation). The mutagenic effect of AL extract and both compounds on both CCA cells and normal cells was shown to be concentration-dependent. Following exposure to the three test materials for 24 hours, the number of micronucleus was increased in both cell lines compared to untreated cell. In addition, the decrease in micronucleus number was observed with low concentrations of these test materials compared with positive control. Results suggested weak mutagenic effects of AL ethanolic extract and its bioactive compounds in all cell lines investigated. The observation confirms the previous report of mutagenesis in CCA which may complicate CCA chemotherapy [75]. This finding required further confirmation in additional* in vitro* as well as* in vivo* models particularly following long-term exposure.

### 7.4. Apoptotic Activities and Molecular Targets of Action of *β*-Eudesmol and Atractylodin in Cholangiocarcinoma

Apoptosis plays an important role for multicellular organisms to maintain tissue homeostasis and eliminate unwanted cells [76]. To evade apoptosis is one of the most characterized features of tumor cells [77]. Currently, killing tumor cells via apoptosis is becoming a most potential and attractive strategy for cancer prevention and treatment. The aim of this part of the study was to preliminarily investigate the inhibitory effects of AL extract and the two major bioactive compounds, *β*-eudesmol and atractylodin, on human CCA (CL-6) cell growth inhibition (MTT-based cytotoxic assay), cell survival, cell migration, cell cycle arrest, and cell apoptosis [78]. The underlying mechanisms/targets through which *β*-eudesmol and atractylodin exert their effects on heme oxygenase-1 (HO-1) production, STAT1/3 phosphorylation, and NF-*κ*B protein expression were also examined.

The cytotoxic activities of both compounds were concentration- and time-dependent. The IC_50_ (mean ± SD) of atractylodin and *β*-eudesmol against CL-6 were 41.66 ± 2.51 and 39.33 ± 1.15 *μ*g/ml, respectively. Inducing effects of AL extract on CL-6 cell apoptosis were 5.50%. The effect on normal cell (OUMS-36T-1F) was 6.38%. Induction of apoptosis by *β*-eudesmol occurred only in CL-6 cell (37.84%), but not in OUMS-36T-1F cell. In contrast, induction of apoptosis by atractylodin was observed in both cells [27.21% and 31.74% for CL-6 and OUMS-36T-1F cells, resp.].

The induction of cell cycle arrest was observed at G1 phase, and the potent induction of cell apoptosis was detected at 48 hours of exposure to both compounds. *β*-eudesmol treatment suppressed colony forming and wound closure ability of CL-6 in a concentration-dependent manner. Western blot analysis indicated that *β*-eudesmol and atractylodin treatment resulted in insignificant suppression of HO-1 production in CL-6 cells. Both inhibited STAT1/3 protein phosphorylation and downregulated NF-*κ*B (p65 and p50) proteins expression in a concentration-dependent manner. Taken together, these results suggest that *β*-eudesmol and atractylodin exert significant growth inhibitory activity on CL-6 cells which may be linked to their inhibitory activities on HO-1 production, STAT1/3 phosphorylation, and expression of major NF-*κ*B proteins. Detailed investigations of the molecular mechanisms of cytotoxicity of both compounds in human CCA cells are underway.

### 7.5. Pharmacokinetics of *β*-Eudesmol in Mice

**Figure 9:**
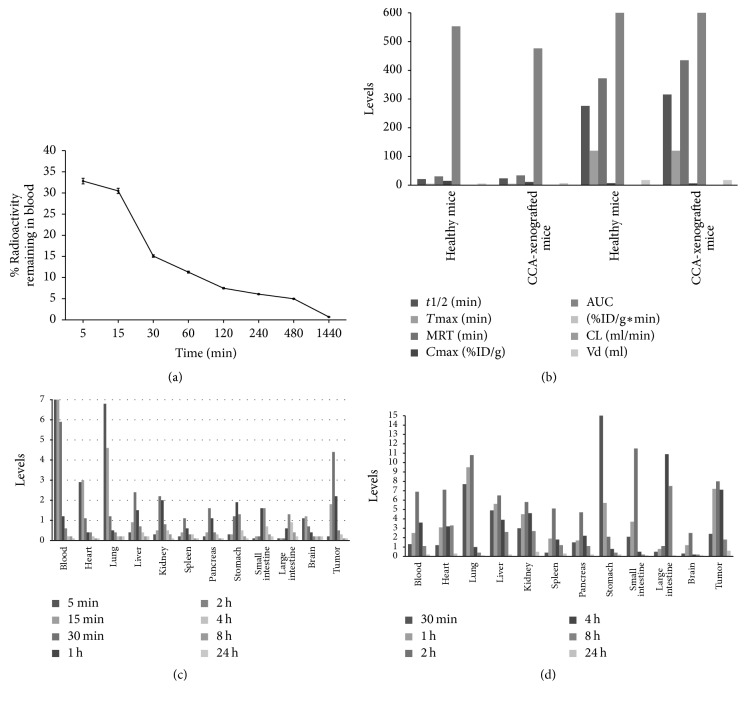
Blood clearance (a), pharmacokinetic profiles (b) of ^99m^Tc- *β*-eudesmol in healthy mice and tissue distribution of *β*-eudesmol following intravenous (c), and oral (d) dosing of ^99m^Tc- *β*-eudesmol in CCA-xenografted mice.

PET-CT with radiolabel ^99m^Tc was applied for monitoring of blood and tissue kinetics of *β*-eudesmol in healthy and CCA-xenografted nude mice following oral and intravenous dose administration [27]. Oral absorption of *β*-eudesmol was relatively rapid with about 70–73%, 25–31%, and 78–81% of the administered dose being detected in stomachs, small intestines, and large intestines of both groups of mice. Systemic clearance of *β*-eudesmol was rapid with complete elimination in urine and feces within 48 hours of dosing. The compound distributed to most vital organs at maximum levels 2 hours after oral administration and 15 minutes after intravenous administration (Figure 9). The pharmacokinetics of ^99m^Tc-*β*-eudesmol complex following intravenous and oral administration was influenced by CCA. Significant difference in the pharmacokinetics was also observed between oral administration and intravenous injection, which was explained by difference in the bioavailability of ^99m^Tc-*β*-eudesmol from each administration route and dosage form. The mean residence time of the complex (median values of MRT: 30.53 versus 33.92 min and 372.11 versus 435.03 min for healthy versus CCA-xenografted mice following intravenous and oral administration, resp.) was significantly prolonged mainly as a consequence of accelerated systemic clearance (median values of CL: 0.18 versus 0.21 ml/min for healthy versus CCA-xenografted mice following intravenous administration, respectively). This kinetic change together with the expansion of apparent volume of distribution (median values of Vd: 5.52 versus 7.12 ml for healthy versus CCA-xenografted mice following oral administration) resulted in a significant decrease in systemic bioavailability expressed as area under serum concentration-time profile (median values of AUC: 553.42 versus 476.34 %ID/g*∗*min and 2,176.50 versus 2,428.50 %ID/g*∗*min for healthy versus CCA-xenografted mice following intravenous and oral administration, resp.).

### 7.6. Potential of Metabolic Drug Interaction

Herbal medicines are mixtures of more than one active ingredient, and, therefore, the likelihood of drug-herb interaction is theoretically higher than drug–drug interaction as synthetic drugs usually contain single chemical entities. One of the most important mechanisms through which interactions occur is via the interference of herbal products with human hepatic drug metabolizing enzyme activities particularly the cytochrome P450 (CYP) enzymes. This interaction could lead to the reduced metabolism of coadministered drugs which are CYP substrates, leading to potential risks of toxicity. Among all human CYPs, CYP3A4 is reported to be involved in the metabolism of 50% of all pharmaceuticals [79]; it is therefore an enzyme of general interest for herb–drug interactions. Several herbs and natural remedies have been reported to induce or inhibit CYP3A4. Previous studies have demonstrated potential CYP inhibitory effects of herbal medicines including grape fruit juice [80, 81], garlic [82, 83], ginseng (Ginseng spp.) [84], soya bean (Glycine max) [84], black cohosh [85, 86], St. John's wort [87–89], Echinacea [87–89], and common valerian [87].

The propensity to inhibit CYP1A2, CYP2C19, CYP2D6, and CYP3A4 of the AL extract was investigated using human liver microsomes [90]. Results showed potent inhibitory activity of AL on CYP1A2-mediated phenacetin O-deethylation with mean IC_50_ of 0.36 *μ*g/ml (36-fold of the inhibitor *α*-naphthoflavone). Inhibition of CYP1A2 by AL extract should be beneficial to CCA prevention and/or therapy in view of the contribution of CYP1A2 in carcinogenesis of CCA [91]. The potency of inhibitory effects on CYP3A4, CYP2D6, and CYP2C19 was low (mean IC_50_ 313.51, 54.36, and 16.48 *μ*g/ml, resp.). Based on these data, clinical use of AL as crude extract should be safe with respect to its low propensity to interfere with major hepatic CYP metabolism when given together with other drugs particularly as an adjunctive therapy with conventional chemotherapeutic drugs.

## 8. Development of Oral Pharmaceutical Formulation of Standardized* Atractylodes lancea *(Thunb) DC. Extract for Large Scale Production

Based on the previously described results, particularly results of the* in vitro* synergistic interaction between components of AL extract, it is decided that further development of AL should be as crude AL extract. The purpose of this part of study was, therefore, to develop oral pharmaceutical formulation of the standardized AL extract for large scale production (manuscript in preparation). Dried rhizomes of AL (100 kg) obtained from the Charoensuk Osod herbal pharmacy in Nakhon Pathom province was macerated with 95% ethanol 24 hr twice according to the method described in Section 6.2. *β*-eudesmol and atractylodin were used as marker compounds for quality control of the AL extract. The dried AL extract was formulated in one capsule (No. 00) with lactose (water-soluble filler), sodium lauryl sulfate (surfactant), and talcum (glidant) at the ratio of ratio of 3 : 1 : 0.0005 : 0.1. The pharmaceutical properties of the AL capsule were evaluated using standard procedures (US Food and Drug Administration). Results showed acceptable properties of the formulation (bulk density, solubility, tapped density, Hausner ratio, compressibility index, angle of repose, flow ability, weight variation, disintegration, and dissolution). Cytotoxic activity of the oral pharmaceutical of standardized AL extract against CL-6 cells was confirmed using MTT assay and result showed similar activity of the crude ethanolic extract (mean ± SD IC_50_  29.60 ± 2.24 *μ*g/ml).

## 9. Toxicity Testing of Oral Pharmaceutical Formulation of Standardized* Atractylodes lancea *(Thunb) DC. Extract

Acute (single oral dose) and subacute (daily oral doses for 28 days) toxicity testing of the oral pharmaceutical formulation of the standardized AL extract were carried out in Wister rats to confirm the toxicity profile, MTD and NOAEL levels of the formulated AL as previously observed initially with the crude ethanolic AL extract [25]. The experiment was carried out as previously described in Section 5.2. Results were similar to that observed with the crude ethanolic AL extract showing virtually no toxicity up to the highest recommended dose of 5,000 mg/kg body weight (single and repeated doses). Only mild signs of acute toxicity were observed, that is, stomach irritation and general CNS depressant signs (reduced alertness and locomotion and diminished response to touch and balance) which occurred within 1 hour after feeding with highest dose (5,000 mg/kg body weight). All were reversible and recovered within 24 hours of dosing. The MTD and NOAEL levels of the oral pharmaceutical formulation of standardized AL extract were 5,000 mg/kg body weight.

## 10. Conclusions and Way Forward

The modern drug discovery processes have started revisiting traditional knowledge and ethnopharmacology to reduce the innovation deficit faced today that would help reach to the top in sciences. To be accepted as viable alternative to modern medicine, however, the same vigorous method of scientific and clinical standards must be applied to prove the safety and effectiveness of a therapeutical product. Traditional herbal products are heterogeneous in nature. They impose a number of challenges to qualify control, quality assurance, and the regulatory process. They should be subjected to drug approval process to demonstrate their safety and effectiveness. Sometimes, patients use traditional and conventional medicine simultaneously. The interaction of these two types of drugs* in vivo* may be dangerous and has raised serious concern among the medical scientists about the safety of the patients. This article reviews steps of research and development of* A. Lancea *(AL) as potential candidate for CCA chemotherapy, with adoption of the reverse pharmacology concept, to ensure that the path of development is in compliance with applicable quality standards and regulatory requirements. Reverse pharmacology is considered as a fast tract path of the discovery of drugs through natural products. Extensive past experiences in the traditional uses of AL accelerates and in addition, reduces the cost in drug development process through short-cutting the discovery step. The AL extract was directly screened for cytotoxic activity against CCA cells. Its long traditional use of AL provides strong evidence for its safety profile in humans which facilitates dose optimization. This available knowledge on AL was used as a starting point for development of AL for chemotherapy of AL before the first human trial. Results from a series of our study confirm anti-CCA potential and safety profiles of both the crude extract and the finished product (oral pharmaceutical formulation of the standardized AL extract. Relevant quality standards were applied throughout all various steps. The concept of “Good Clinical Laboratory Practice (GCLP)” was applied for all* in vitro* nonclinical studies. The quality standard “Good Laboratory Practice (GLP)” was applied to toxicology studies of the finished product for evaluating acute and subacute toxicity in animals. In further step, large scale production of the oral pharmaceutical formulation of the standardized AL extract is being prepared under “Good Manufacturing Practice (GMP)” standard. This will follow soon by phase I and Phase II clinical trials. The basic ethical and scientific principles of research in human subjects “Good Clinical Practice (GCP)” used in the modern medicine will be applied in both clinical trial phases. The purpose of the Phase I study in healthy subjects is mainly to evaluate tolerability profile of the oral pharmaceutical product using the first-starting dose in human. The first-starting dose of the AL extract in human will be estimated based on the maximum recommended starting dose (MRSD) as the ratio of human equivalence dose (HED) and (Safety factor × [animal weight (kg)/human weight (kg)]^0.33^) [92]. The HED will be estimated based on NOAEL of AL extract obtained from the most sensitive species (mouse) as described in Section 9. In addition, the study is planned to investigate pharmacokinetics of the two bioactive constituents, *β*-eudesmol and atractylodin. The phase II clinical study is aimed at the target population of patients with advanced stage CCA in the northeastern region of Thailand to evaluate clinical efficacy and safety of the oral pharmaceutical product when given alone and in combination with standard treatment (cisplatin and gemcitabine combination).

Apart from the clinical investigations, parallel nonclinical studies will also be conducted to fully explore the underlying molecular mechanisms of action of AL in CCA. As herbal medicines usually contain a range of pharmacologically active compounds, their multi-ingredient character can render efficacy testing more complex than with synthetic drugs. On the other hand, using the whole plant extract would be expected to provide therapeutic benefit compared to isolated compounds with respect to efficacy (synergistic action) and tolerability (“buffering effect”). In this scenario, current strategy of drug development of single target single compound based on a super reductionism that involves molecular level assays may not be suitable for studies on herbal medicines. A more holistic approach using systems biology through the application of metabolomics techniques appears much more suited to probe and confirm the efficacy and understand the mode of action. Metabolomics is a systems approach for studying metabolic profiles, which promises to provide information on drug toxicity, disease processes, and gene function at several stages in the discovery process. Furthermore, investigation of immune-modulatory effect of the plant extract/bioactive constituents will be explored both* in vitro* and* in vivo*. The contribution of immune-modulatory effects of various plants on cancers has been well demonstrated [93–98].
